# Identification of the salusin-β receptor using proteoliposomes embedded with endogenous membrane proteins

**DOI:** 10.1038/s41598-018-35740-6

**Published:** 2018-12-14

**Authors:** Masayoshi Shichiri, Daisuke Nonaka, Lyang-Ja Lee, Kenji Tanaka

**Affiliations:** 1 Department of Endocrinology, Diabetes and Metabolism, Kitasato University School of Medicine, 1-15-1 Kitasato, Minami-ku, Sagamihara, Kanagawa 252-0374 Japan; 2 Protosera Inc., 4-3-22 Nishinakajima, Yodogawa-ku, Osaka, 532-0011 Japan

## Abstract

Although orphan G protein-coupled receptors (GPCRs) have been used as targets to discover unidentified natural ligands, increasing numbers of non-GPCRs have been found to mediate important biological functions. Bioinformatics of genome and cDNA resources predict putative bioactive peptides, demanding an alternative approach to efficiently unravel cell surface targets. *In silico* analysis of a full-length cDNA library previously allowed us to identify salusin-β, a parasympathomimetic/pro-atherosclerotic peptide with unique physicochemical properties. Here, we show that the β-chain of ATP synthase is a cell surface receptor for salusin-β by utilizing artificial liposomes embedded with endogenous membrane proteins directly transferred from animal tissues while retaining the ligand-binding capability. Conventional techniques using detergents identified a β-actin-profilin complex as membrane-associated salusin-β-binding proteins, but failed to identify the cell surface receptor. Since the α-chain of ATP synthase is a principal cell surface target for angiostatin, a potent endogenous angiogenesis inhibitor, we investigated whether salusin-β modulates angiogenesis. Salusin-β inhibited cell surface ATP synthase activity and prevented sarcoma cell-induced angiogenesis in an *in vivo* mouse air sac model. Therefore, salusin-β binds to membrane-bound ATP synthase and acts as an angiogenesis inhibitor. The current methodology allows the identification of novel cell surface targets, irrespective of the receptor structure.

## Introduction

An approach using orphan G protein-coupled receptors (GPCRs) as targets to isolate their ligands, also termed reverse pharmacology, has allowed the identification of novel bioactive peptides^1^. However, the numbers of deorphanized GPCRs and newly identified transmitters have drastically decreased during the past decade, and the search for new ligands and their receptors is encountering major difficulties^2^. The approach relies on monitoring changes in currently known second messenger levels and on utilizing the limited pool of known transmitters. Although the use of bioinformatics has helped to identify bioactive peptides by a reverse pharmacology approach^3–6^, our more comprehensive *in silico* analysis of a full-length enriched cDNA library taking advantage of structural features, such as possession of signal sequences and being flanked by processing sites, has successfully identified novel bioactive peptides, such as salusin-β^7,8^. Further, our recent success in comprehensive identification of plasma low-molecular-weight native peptides using LC-MS/MS after efficient removal of high abundant plasma proteins^9,10^ is now engendering “orphan ligands”. Endogenous bioactive peptides may utilize non-GPCRs, while other peptides traditionally optimized for a single receptor were later shown to act on multiple receptors, many of which are non-GPCRs. Since such “off targets” may later be shown to have hidden bioactivities, an alternative approach that replaces reverse pharmacology and can efficiently identify cell surface targets is required.

A number of membrane proteins have previously been embedded in artificial liposomes; however, identifying an unknown receptor using endogenous membrane protein-embedded liposomes still remains extremely challenging. Proteoliposome techniques using detergents to extract membrane proteins increases the difficulty of *in vitro* approaches^11,12^, with detergents affecting the ligand binding capabilities of reconstituted proteoliposomes^13,14^. Moreover, conventional proteoliposomes can aggregate with one another and are difficult to pass through affinity columns. Such insoluble proteoliposomes cannot even be applied to receptor-binding experiments for subsequent affinity purification of the bound complexes. Moreover, large proteoliposomes with multilamellar structures enclose the great majority of cell membrane proteins within multilayered liposomes, which is another major obstacle to efficiently retrieve highly purified receptor proteins.

We have successfully overcome these limitations and advanced our novel proteoliposome methodology to identify cell surface receptors. First, our protocol allowed us to prepare membrane protein-liposome complexes without the use of detergents. Second, we successfully emulsified our proteoliposomes by size selection, and they never precipitated during the entire processes of binding experiments and affinity purification. Third, we determined suitable protein-lipid ratios for constituting proteoliposomes to achieve fewer receptor proteins expressed per liposome particle. This enrichment process is essential for purifying target receptor proteins that can be analyzed by mass spectrometry. These technical advances enabled the transfer of entire endogenous membrane proteins into emulsified artificial liposomes and the retrieval of target receptors utilizing immobilized peptides.

These results prompted us to design a study to identify the cell surface receptor for salusin-β, which exerts potent hypotensive, bradycardic, antidipsogenic and pro-atherosclerotic effects^8,15–18^.

## Results

### Emulsification of liposomes embedded with endogenous membrane proteins

**Figure 1:**
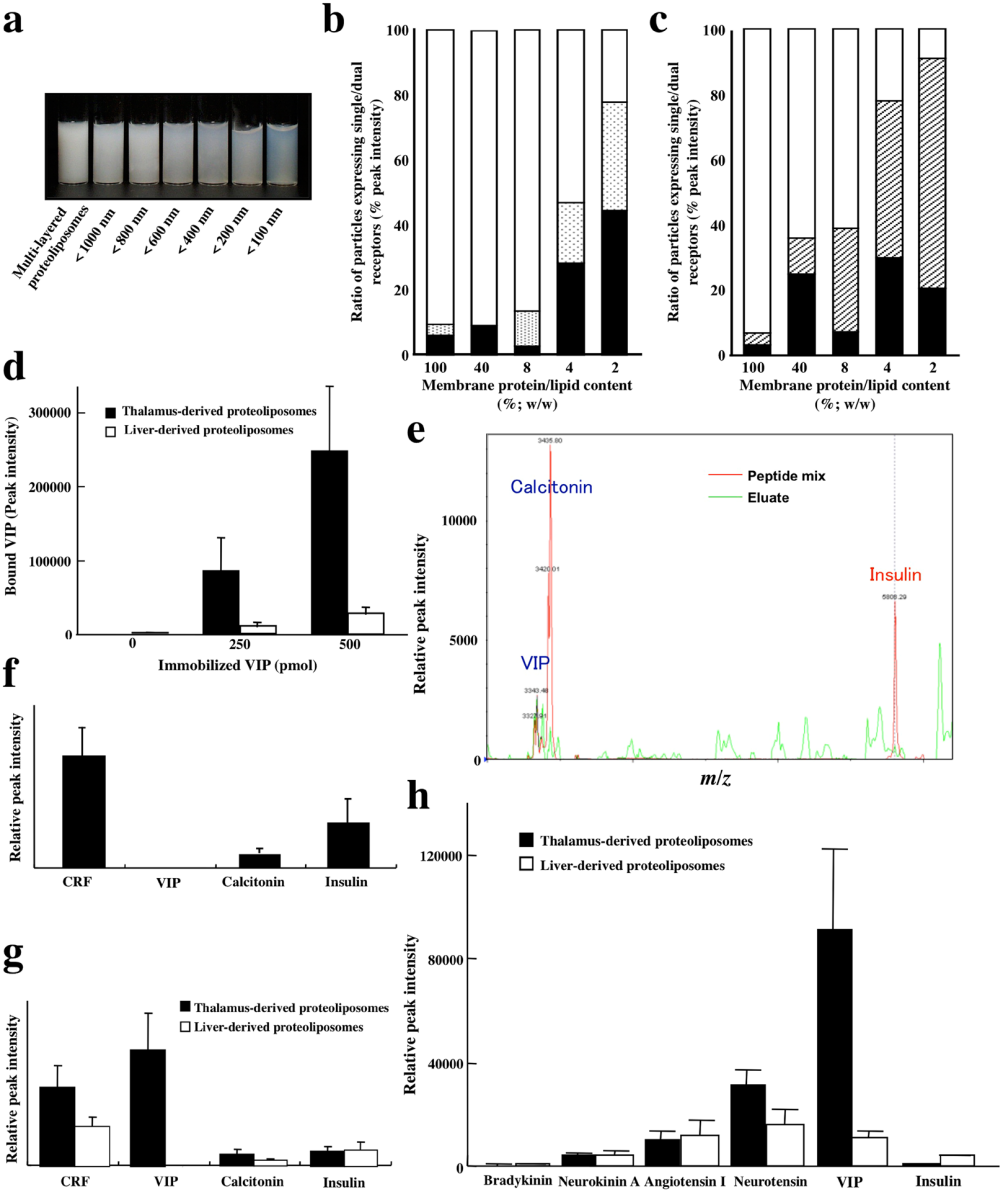
Ligand-binding capabilities of particle-sized liposomes embedded with endogenous membrane proteins. (**a**) Emulsification of membrane proteins-embedded liposomes by particle sizing. Membrane proteins prepared from cultured U937 cells were embedded into liposomes (left tube) and size-selected crudely by passing the liposomes through polycarbonate membranes of the indicated pore diameters. (**b**,**c**) Effects of the membrane protein-lipid ratio used for constituting proteoliposomes on the ratio of proteoliposomes expressing a single uPA receptor (closed column) or C5a (dotted column)/interferon-γ receptor (hatched column) *vs*. those expressing dual receptors (open columns) on the surface of the liposomes. Membrane proteins were embedded into liposomes at the indicated membrane protein-lipid ratios. After particle sizing, the numbers of proteoliposomes expressing single or dual receptors were determined by flow cytometry analysis. Membrane protein fractions directly prepared from cells were also analyzed by flow cytometry for comparison (left columns). (**d**) Dose-dependent retrieval of VIP peptide bound to its receptor on the surface of liposomes. Synthetic VIP was allowed to bind to liposomes embedded with membrane proteins prepared from porcine thalamic and hepatic tissues. Bound VIP peptides were eluted and subjected to mass spectrometry analysis. (**e**) Retrieval of a peptide mixture bound to thalamus-derived proteoliposomes. A mixture of VIP, calcitonin and insulin was reacted with proteoliposomes derived from porcine thalamus and the molecular masses of both the eluates (green line) and the control peptide mixture (red line) were determined. (**f**) Retrieval of CRF and other peptides bound to proteoliposomes prepared from CRF receptor-expressing HEK293 cells. Membrane protein-embedded liposomes prepared using HEK293 cells that had been transfected with a CRF receptor cDNA were allowed to bind to a mixture of the indicated peptides. The eluate was subjected to mass spectrometry analysis. (**g**,**h**) Retrieval and detection of peptide mixtures bound to proteoliposomes derived from porcine thalamus and liver tissues. Mixtures of the indicated peptides were reacted with liposomes embedded with membrane proteins prepared from porcine thalamic and hepatic tissues and the eluates were analyzed by mass spectrometry.

We previously created a protocol utilizing artificial liposomes embedded with innate cell membrane proteins that had been directly transferred from animal tissues or cultured cells^19^. Cell membrane fractions extracted from cultured human monocytic U937 cells were mixed with liposomes consisting of purified yolk lecithin and cholesterol, and membrane protein-embedded liposomes were prepared. Such proteoliposomes consisted of 100–5000 nm diameter particles and contained many large multilayered liposomes that aggregated with one another. Removing the large particles using a 200-nm polycarbonate membrane reduced the aggregation and led to emulsification of the membrane proteins-embedded liposomes (Fig. 1a). Proteoliposomes of <200 nm were emulsified and never precipitated unless subjected to ultracentrifugation. Using such particle-sized proteoliposomes, we determined whether reductions in the protein-lipid ratio of the liposomes changed the rate at which multiple receptors were expressed compared with fewer receptors on the surface of the proteoliposomes. Urinary plasminogen activator (uPA) receptor is a 50-kDa multidomain glycoprotein tethered to the cell membrane with a glycosylphosphatidylinositol (GPI) anchor. The human monocytic cell line U937 expresses receptors for uPA, interferon-γ and serum complement component C5a, which are markedly upregulated by stimulation with phorbol 12-myristate 13-acetate (PMA). Flow cytometric analysis revealed that as the membrane protein-lipid ratio decreased, the numbers of proteoliposomes expressing either a C5a receptor or uPA receptor increased while those expressing both C5a and uPA receptors decreased (Fig. 1b). Similarly, reductions in the protein-lipid ratio increased the percentages of proteoliposomes expressing either interferon-γ receptor or uPA receptor and reduced those expressing both receptors (Fig. 1c). We concluded that liposomes containing a 2% (w/w) membrane protein-lipid content should be used for embedding membrane proteins and that proteoliposomes of less than 200 nm after particle-sizing should be used for receptor retrieval experiments.

### Ligand-binding capabilities of membrane proteins-embedded liposomes

Next, we repeated the binding experiments to confirm whether the receptor proteins incorporated into liposomes using the above-described protocol retained their binding affinity toward a variety of ligands. The vasoactive intestinal peptide (VIP) receptor VPAC_1_ is a GPCR that is abundantly expressed in the brain, liver, lung and intestine. Membrane proteins prepared from porcine thalamus and liver tissues were embedded into liposomes and allowed to bind to various doses of VIP. Each eluate contained a single molecular mass of VIP and the thalamus appeared to bind to a greater amount of VIP per tissue weight than the liver (Fig. 1d). Three synthetic peptides, calcitonin, VIP and insulin, were mixed and subjected to binding experiments with thalamic tissue-derived proteoliposomes. Mass spectrometry of the reaction eluate revealed peaks coinciding with the three peptides (Fig. 1e). We transfected HEK293 cells with a corticotropin-releasing factor (CRF) receptor cDNA, prepared membrane proteins-embedded liposomes, and allowed them to bind to a mixture of synthetic CRF, VIP, calcitonin and insulin. Mass spectrometry of the eluate showed peaks corresponding to CRF, calcitonin and insulin, but not VIP (Fig. 1f), whereas untransfected cells did not show any binding to CRF (data not shown). These data demonstrate that the method allows retrieval of CRF receptors transfected into HEK293 cells, in addition to endogenous calcitonin and insulin receptors. Thalamic tissue-derived proteoliposomes had abundant binding sites for VIP and CRF, and fewer binding sites for calcitonin and insulin, whereas hepatic tissue-derived proteoliposomes bound most preferentially to CRF, less preferentially but still significantly to calcitonin and insulin, and negligibly to VIP (Fig. 1g). Thalamic and hepatic tissue-derived proteoliposomes were reacted with a mixture of bradykinin, neurokinin A, angiotensin I, neurotensin, VIP and insulin. All the ligands were retrieved in the eluates, as demonstrated by mass spectrometry (Fig. 1h). Therefore, the current method allows simultaneous detection of different types of receptors using a mixture of peptide ligands. Taken together, these results indicate that multiple ligands binding to membrane proteins-embedded liposomes can be identified as long as the molecular weights of the ligands and receptors allow separation by current mass spectrometry technology.

### Retrieval of cell surface receptors using membrane proteins-embedded liposomes

**Figure 2:**
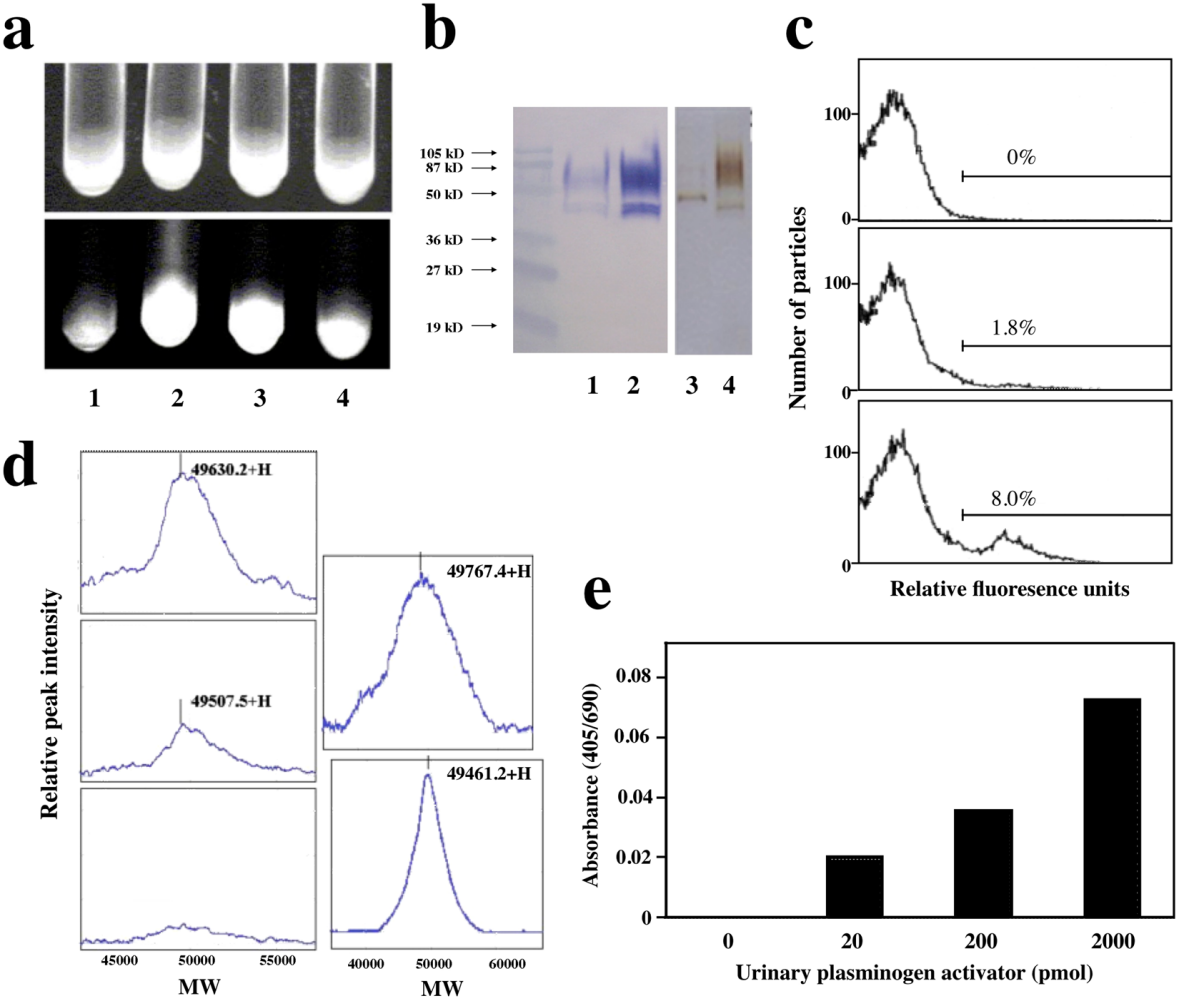
Isolation and detection of urinary plasminogen activator (uPA) receptor using liposomes embedded with membrane proteins derived from cultured human cells. (**a**) Binding of uPA, interferon-γ and complement C5a to their respective receptors embedded into fluoroliposomes. Membrane proteins prepared from PMA-stimulated U937 cells were embedded into FITC-labeled liposomes, mixed without (1) or with biotinylated uPA (2), interferon-γ (3) or complement C5a (4) immobilized on an avidinylated Sepharose 4B gel. After extensive washing, the liposomes were observed under visible light (upper panel) and fluorescent light (lower panel). (**b)** Western blot of uPA receptor and uPA proteins. (**a**) proteoliposome fraction (lane 1) and a conventional membrane protein fraction (lane 2) were prepared from PMA-stimulated U937 cells and detected with a specific antibody against uPA receptor. Purified uPA (lane 3) and purified uPA receptor (lane 4) were also observed after silver staining. (**c**) Flow cytometric determination of uPA receptors in membrane protein-embedded liposomes using FITC-labeled uPA. FITC-labeled human serum albumin, a negative control, does not bind to proteoliposomes prepared using PMA-stimulated U937 cells (upper panel), whereas 1.8% and 8.0% of the proteoliposomes derived from PMA-unstimulated (middle panel) and PMA-stimulated (lower panel) U937 cells exhibit binding to FITC-labeled uPA, respectively. (**d**) Mass spectrometric detection of uPA and its receptor. Protein G covalently bound to the plate for mass spectrometry was used to bind anti-uPA IgG, and purified uPA was then applied to the same plate. Membrane proteins-embedded liposomes prepared from PMA-stimulated U937 cells (upper left panel) or simple liposomes without membrane proteins (middle left panel) were overlaid on the plate and subjected to mass spectrometric analysis. Control experiments were carried out with bovine IgG in place of anti-uPA IgG (bottom left panel). Mass spectrometry of purified uPA receptor and uPA proteins (upper and lower right panels). (**e**) Dose-dependent retrieval of uPA receptor from membrane proteins-embedded liposomes. Membrane proteins derived from PMA-stimulated U937 cells were embedded into liposomes containing alkaline phosphatase and reacted with the indicated doses of purified uPA immobilized on Sepharose 4B. Bound complexes were lysed in a buffer containing Triton X-100, followed by the addition of an alkaline phosphatase substrate and measurement of the absorbances.

Liposomes labeled with fluorescein-isothiocyanate (FITC) were embedded with membrane proteins prepared from PMA-stimulated U937 cells, and reacted with biotinylated uPA, interferon-γ or C5a immobilized on an avidinylated Sepharose 4B gel as a ligand support. After removing unbound fluoroliposomes by extensive washing, only gels containing receptor-bound ligands emitted fluorescence under ultraviolet light in the dark field (Fig. 2a), suggesting binding of each ligand to its respective receptor on the liposome surface. Immunoblotting with a specific antibody demonstrated that uPA receptor proteins isolated using proteoliposomes exhibited a similar electrophoresis pattern to conventionally isolated uPA receptor and purified uPA receptor proteins in SDS-polyacrylamide gels (Fig. 2b). Binding experiments using fluorescent peptides revealed that FITC-labeled uPA, but not FITC-labeled human serum albumin, bound to liposomes embedded with membrane proteins, as demonstrated by flow cytometry analysis (Fig. 2c, upper and middle panels). Stimulation of U937 cells with PMA prior to membrane protein preparation markedly enhanced the binding of FITC-labeled uPA (1.8–8.0%) to the proteoliposomes (Fig. 2c, lower panel), suggesting that upregulated uPA receptors were transferred to liposomes and retained their ligand-binding capability. Next, we confirmed the retrieval of uPA receptors on the surface of the proteoliposomes using a uPA peptide covalently crosslinked to a plate for mass spectrometry. The proteoliposome-uPA reaction complex on the plate revealed a peak (Fig. 2d, upper left panel) between purified uPA (Fig. 2d, lower right panel) and its receptor protein (Fig. 2d, upper right panel), corresponding to a summation peak consisting of both uPA and its receptor. Control experiments using empty liposomes and uPA produced a single peak corresponding to purified uPA (Fig. 2d, middle left panel). Next, we embedded U937-derived membrane proteins into liposomes containing alkaline phosphatase, performed binding experiments with immobilized uPA and lysed the eluted proteoliposomes in a buffer containing Triton X-100. The resultant alkaline phosphatase activity quantitatively reflected the amount of uPA receptor isolated from the proteoliposomes (Fig. 2e). These results demonstrate that the current method could be applicable to isolating GPCRs, GPI anchors and oligomer-type receptors.

### Binding experiments and retrieval of salusin-β from membrane proteins-embedded liposomes

**Figure 3:**
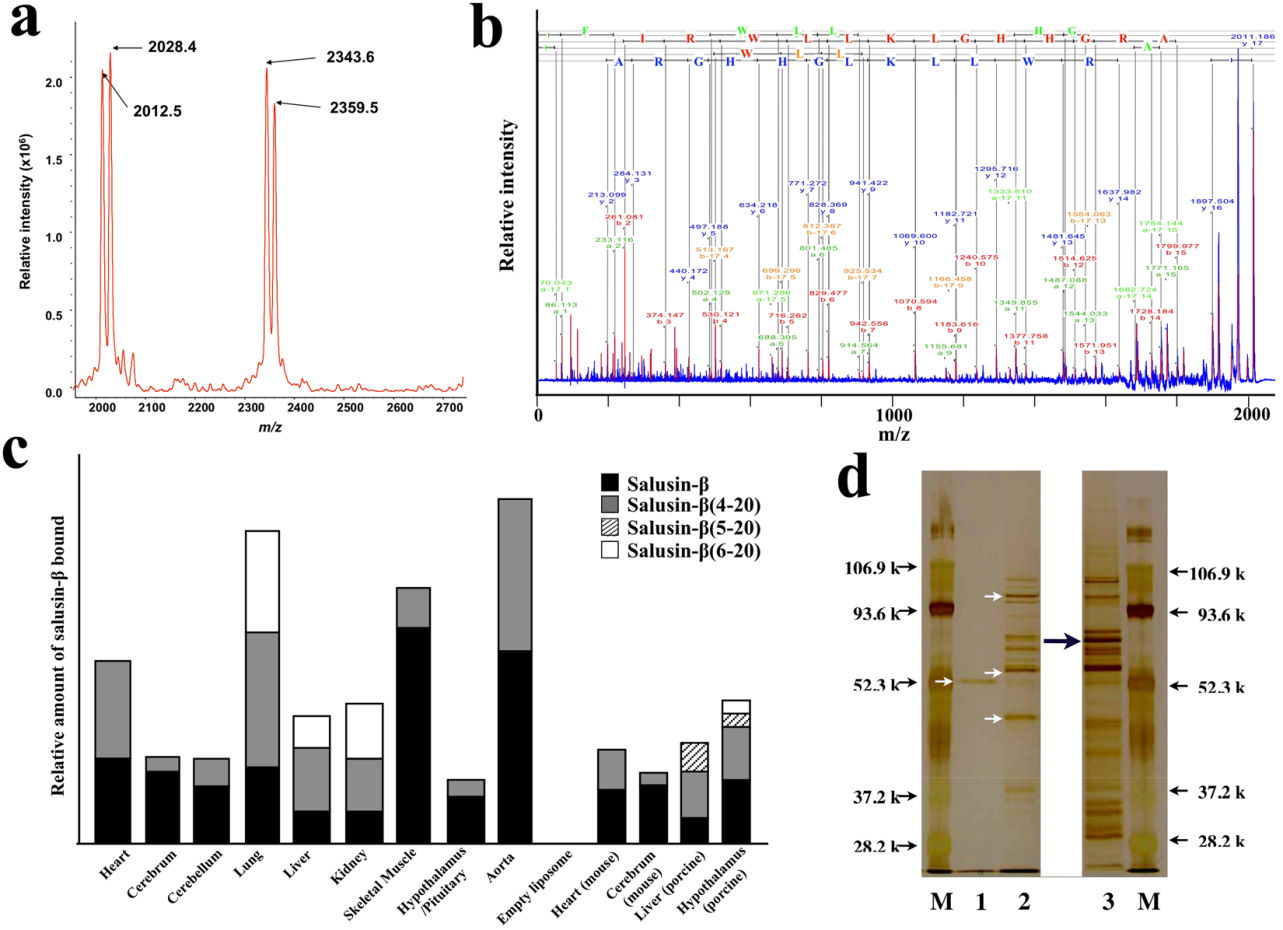
Affinity purification of salusin-β-binding proteins/receptor. (**a**) Truncation of the N-terminal three amino acids of salusin-β after binding to membrane proteins-embedded liposomes. Synthetic salusin-β (500 pmol) was allowed to bind to liposomes embedded with membrane proteins prepared from rat cardiac tissues and subjected to MALDI-MS analysis after elution. (**b**) MALDI-MS/MS spectra revealing truncation of salusin-β to salusin-β(4-20). MS/MS spectra were assigned theoretical m/z values for a, a-H_2_O, and b ions. Parent peptide and MS/MS tolerance parameters were set at ± 10 Da and ± 0.03 Da. (**c**) Retrieval of salusin-β and its truncated forms bound to proteoliposomes prepared from a variety of animal tissues. Membrane proteins-embedded liposomes were prepared from rat, mouse and porcine organs, and binding experiments were performed. The relative amounts of salusin-β (closed column) and its truncated forms (salusin-β(4-20), dark column; salusin-β(5-20), hatched column; salusin-β(6-20), open column) retrieved from the proteoliposomes are expressed in the ordinates. (**d**) Affinity purification of membrane proteins bound to immobilized salusin-β. Lysates containing membrane proteins prepared from rat hearts were subjected to affinity chromatography using C-terminally (lane 1) and N-terminally (lane 2) immobilized salusin-β, and the eluates were separated by SDS-PAGE. Liposomes embedded with membrane proteins prepared from rat heart were allowed to bind to N-terminally immobilized salusin-β (lane 3). Silver-stained bands (arrows) were dissected, hydrolyzed with trypsin and subjected to LC-MS/MS analyses. The electrophoretic mobilities of low molecular weight standards (lane M) are indicated. From the top of the gel these proteins are phosphorylase B (106.9 kDa), bovine serum albumin (93.6 kDa), ovalbumin (52.3 kDa), carbonic anhydrase (37.2 kDa), and soybean trypsin inhibitor (28.2 kDa).

Synthesized salusin-β analyzed by matrix-assisted laser desorption ionization-mass spectrometry (MALDI-MS) was detected as a single *m/z* of 2343.6. When membrane proteins-embedded liposomes prepared from rat cardiac tissues were mixed with 500 pmol of synthesized salusin-β peptide, and bound peptides were eluted from the proteoliposomes and subjected to MALDI-MS analysis, two peaks with *m/z* of 2012.5 and 2028.4 were retrieved in addition to those corresponding to authentic salusin-β and its oxidized form (*m/z* 2359.5) (Fig. 3a). MALDI-MS/MS analysis identified these peaks as salusin-β(4-20) and its oxidized form (Fig. 3b), suggesting that salusin-β may partly undergo N-terminal truncation upon binding to an unidentified membrane protein prepared from the cardiac tissues. Binding experiments were performed using proteoliposomes prepared from various rat organs, mouse heart and cerebrum, and porcine liver and hypothalamus, and the bound ligands were subjected to mass spectrometry analysis. The ligands eluted from the membrane proteins-embedded liposomes from all the tissues contained the cleaved fragment salusin-β(4-0), as well as authentic salusin-β, while the ligands eluted from the proteoliposomes prepared from rat lung, rat and porcine liver, rat kidney and porcine hypothalamus also contained salusin-β(5-20) and/or salusin-β(6-20) (Fig. 3c), suggesting that binding of the salusin-β peptide to proteoliposomes resulted in further N-terminal truncation, irrespective of the tissue origin.

### Isolation of salusin-β-binding membrane proteins using conventional method

To isolate membrane proteins that bind to salusin-β, we immobilized both C- and N-terminal salusin-β to NHS Sepharose 4B gel and ECH Sepharose 4B gel, respectively, since the N-terminal 3–5 amino acids may undergo cleavage upon binding. Salusin-β immobilized on each sepharose gel was first placed in contact with solubilized membrane protein fractions enriched using a detergent^20^. C-terminally immobilized salusin-β generated a single protein with a relative molecular mass of 55 kDa (Fig. 3d, lane 1). By contrast, N-terminally immobilized salusin-β was confirmed to bind to a greater number of membrane proteins, but not to the 55-kDa protein (Fig. 3d, lane 2). Peptide sequences derived from the 55-kDa protein were identical to a segment of the β-actin-profilin complex (chain A), while three major bands retrieved from the N-terminally immobilized salusin-β contained acyl-coenzyme A dehydrogenase, succinate dehydrogenase subunit A, ubiquinol-cytochrome C reductase core protein II, annexin A2 and laminin receptor 1. These data suggest that the conventional method has less chance of retrieving cell surface receptor proteins, and that C-terminally immobilized salusin-β binds to far fewer proteins but is tightly bound to membrane-associated β-actin.

**Figure 4:**
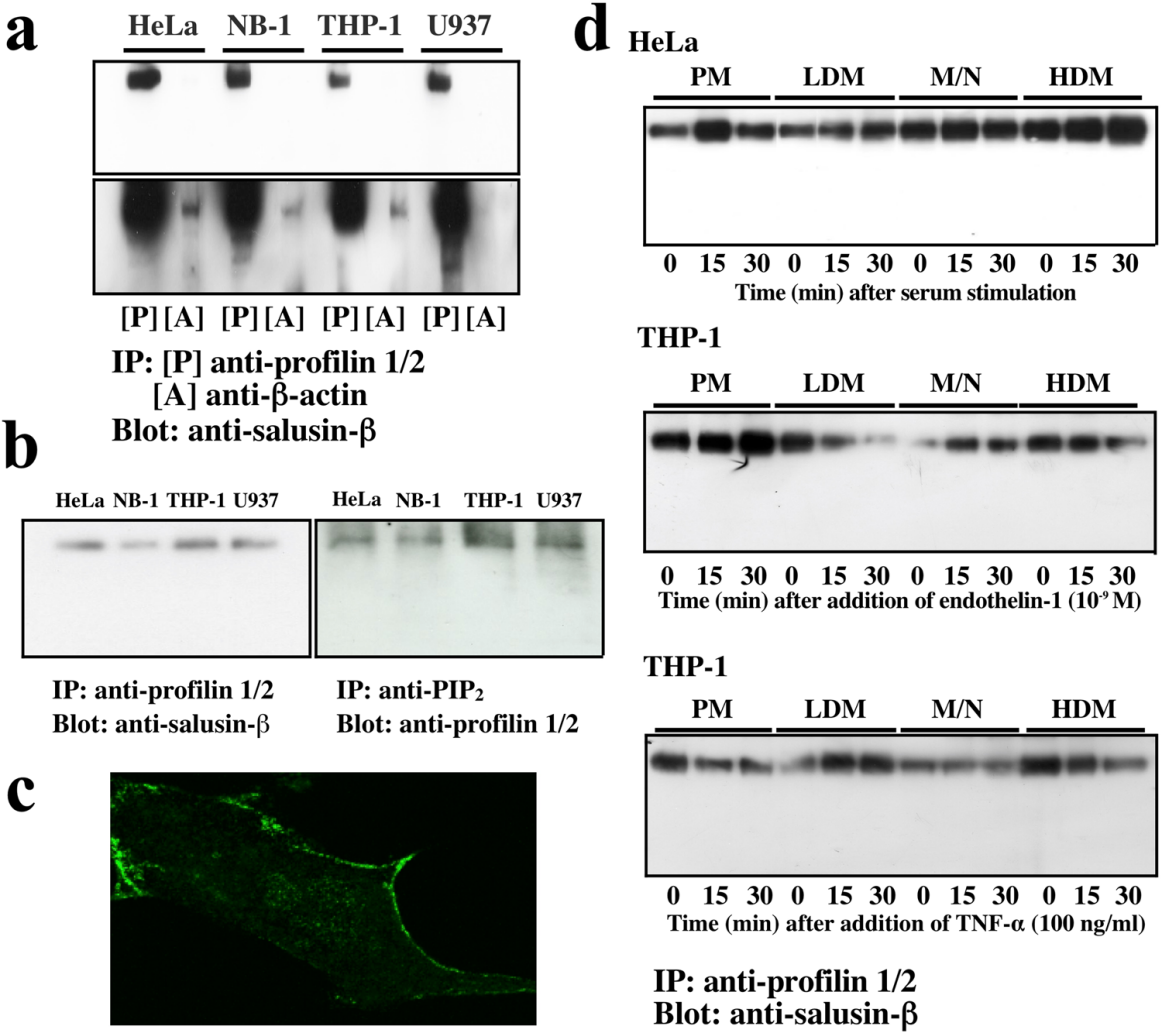
Interaction of intracellular salusin-β with a membrane-associated β-actin-profilin complex. (**a**) Whole cell lysates obtained from the human cell lines HeLa, NB-1, THP-1 and U937 were immunoprecipitated with anti-profilin1/2 or anti-β-actin IgG and immunoblotted with an anti-salusin-β antibody. The upper and lower panels represent 3- and 30-min exposures, respectively, of the same immunoblot. (**b**) Whole cell lysates were immunoprecipitated with anti-profilin1/2 IgG and immunoblotted with the anti-salusin-β antibody (left panel), or immunoprecipitated with anti-PIP_2_ IgG and immunoblotted with anti-profilin1/2 IgG. (**c**) Immunofluorescence staining of NB-1 cells with the anti-salusin-β antibody reveals marked staining in the cell periphery. (**d**) Quiescent HeLa and THP-1 cells stimulated by 10% fetal bovine serum and endothelin-1 (10^−9^ M), respectively (upper and middle panels) and growing THP-1 cells stimulated by TNF-α (100 ng/ml, lower panel) for the indicated times were fractionated by differential centrifugation. Plasma membrane (PM)-, low-density microsome (LDM)-, mitochondria/nucleotide (M/N)-, and high-density microsome (HDM)-enriched fractions were immunoprecipitated with anti-profilin1/2 IgG, separated by SDS-PAGE and analyzed by immunoblotting with the anti-salusin-β antibody.

Because N-terminally immobilized salusin-β was found to bind to the β-actin-profilin complex in our binding experiment using proteoliposomes, we determined whether endogenous salusin-β is associated with the complex in human-derived cell lines. Preincubation of protein extracts from HeLa, NB-1, THP-1 and U937 cells with anti-profilin1/2 IgG or anti-β-actin IgG for immunoprecipitation and subsequent immunoblotting with an anti-salusin-β antibody revealed the presence of intracellular interactions (Fig. 4a). The intracellular salusin-β-profilin complex was also associated with other profilin ligands, such as phosphatidylinositol 4,5-bisphosphate (PIP_2_)^21,22^ (Fig. 4b). Salusin-β-like immunoreactivity was most remarkably detected in the periphery of NB-1 cells (Fig. 4c) and other cells (data not shown). Serum stimulation of HeLa cells increased the amount of the salusin-β-profilin complex in the plasma membrane fraction (Fig. 4d). Endothelin-1 mobilized the salusin-β-profilin complex to the plasma membrane, while the addition of TNF-α reduced the amount of the salusin-β-profilin complex in the cell membrane fraction (Fig. 4d). These data reveal that endogenous salusin-β interacts with the β-actin-profilin complex, and that the dynamic nature of salusin-β is comparable with that of the profilin ligand PIP_2_^23^.

### Identification of a cell surface receptor for salusin-β using membrane proteins-embedded liposomes

**Figure 5:**
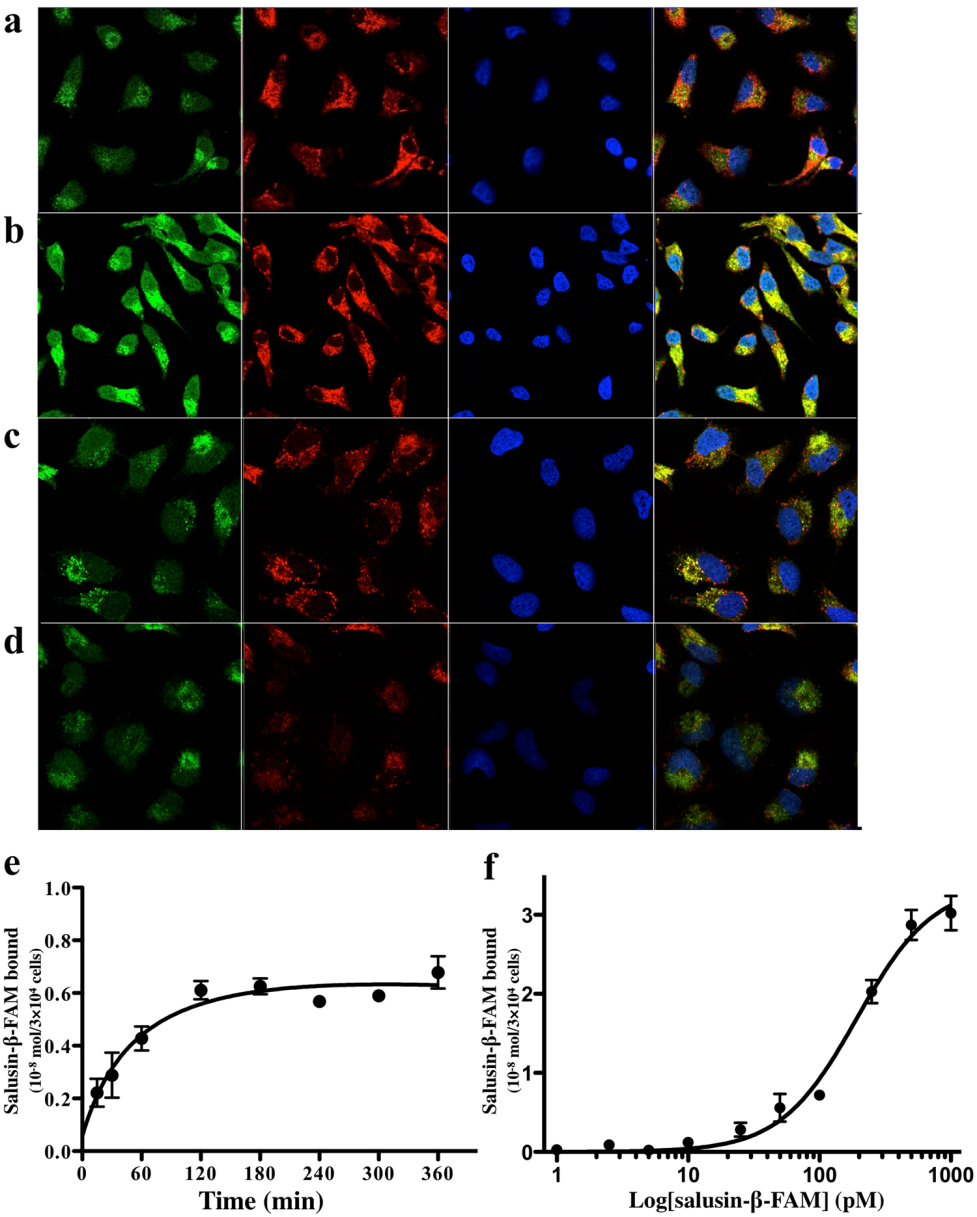
Salusin-β binding to the HeLa cell surface and immunofluorescence colocalization of the ATP synthase β-subunit. (**a**–**d**) Confocal microscopic images of fluorescent salusin-β peptides bound to cultured HeLa cells and immunofluorescent staining for the ATP synthase-β subunit. Salusin-β-FAM (**a**): 10^−8^ M, (**b**–**d**: 10^−7^ M) was overlaid for 1 min on HeLa cells pretreated without (**a**,**b**) or with excess β-casomorphin 7 (**c**) or enterostatin (**d**) for 30 min, fixed and stained with specific antibody against the β-chain of ATP synthase (×1000). The green signals correspond to the cell surface binding sites of salusin-β-FAM. The red signals corresponding to the localization of the β-chain of ATP synthase were obtained with Alexa Fluor^*®*^ 594 secondary antibody (×3000). The nuclei were conterstained with DAPI (blue). Overlay resulted in yellow signals indicative of colocalization. (**e,f**) Quantification of salusin-β-FAM peptide bound to HeLa cell surface. Salusin-β-FAM (10^−6^ M) was overlayed on confluent HeLa cells in 96-well culture plates for the indicated time (**e**) or the indicated concentrations of salusin-β-FAM was added for 180 min (**f**) and, after extensive washing, cell-bound immunofluorescence was measured.

To identify the cell surface target for salusin-β, we performed binding experiments using N-terminally immobilized salusin-β and membrane proteins-embedded liposomes. We excised several bands from the gels and subjected them to trypsin digestion for LC-MS/MS analysis. We found a protein that was only detected in the membrane proteins-embedded liposomes (Fig. 3d, lane 3), and not in the conventional solubilized fraction (Fig. 3d, lane 2). This protein was identified as the β-chain of rat ATP synthase. To determine whether salusin-β binds to the intact cell surface, we incubated several endothelium- and cancer-derived cells with synthetic salusin-β peptide labelled with 5-carboxyfluorescein at either N- (FAM-salusin-β) or C-terminus (salusin-β-FAM). Both FAM-salusin-β and salusin-β-FAM bound to HeLa human cervical carcinoma cell line in a concentration-dependent manner (10^−8^ M–10^−7^ M). Confocal immunofluorescence microscopy with antibodies against the ATP synthase β-subunits revealed specific cell surface signals that were especially strong in the HeLa cells and the signals colocalized very well with those of cells onto which salusin-β-FAM was overlaid to bind to its cell surface receptors (Fig. 5a,b). The binding of salusin-β-FAM to HeLa cells was significantly inhibited by pretreatment of the cells with excess β-casomorphin 7 (Fig. 5c) and enterostatin (Fig. 5d). ATP synthase β-subunit-like immunoreactivity on immunohistochemistry was also reduced by pretreating HeLa cells with excess β-casomorphin 7 (Fig. 5c) and enterostatin (Fig. 5d) before applying antibody against ATP synthase β-subunit. Salusin-β-FAM bound to the surface of confluent HeLa cells in a time- and concentration-dependent manner (Fig. 5e,f). Taken, together, these results demonstrate competitive inhibition of salusin-β binding by these previously identified ligands for the cell surface ATP synthase β-subunit.

Cell surface expression of the ATP synthase β-subunits and binding of FAM-labeled salusin-β was also detected in endothelium-derived cells, such as human umbilical vein endothelial cells (hUVECs) and human dermal microvascular endothelial cells (hMVECs), but was barely detected in endothelial cells originating from larger sized arteries, such as rat aortic endothelial cells (rAECs) or rat pulmonary arterial endothelial cells (rPECs).

### Biological activities mediated by the salusin-β receptor

Salusin-β has a unique physicochemical property that has discouraged many attempts to unravel its biological activity^24^. Purified synthetic salusin-β shows marked adhesiveness to polypropylene, polystyrene and glass and, when reconstituted and diluted in distilled water in such tubes, may rapidly disappear from the solution before being applied to *in vitro* or *in vivo* experiments^14,25,26^. This nature appears to derive partly from the presence of hydrophobic residues at its N-terminal end (Supplementary information), but the problem can be alleviated by the addition of 0.01–0.1% NP40 or Tween 20, thus allowing a fair assessment of its bioactivity^14,26,27^.

**Figure 6:**
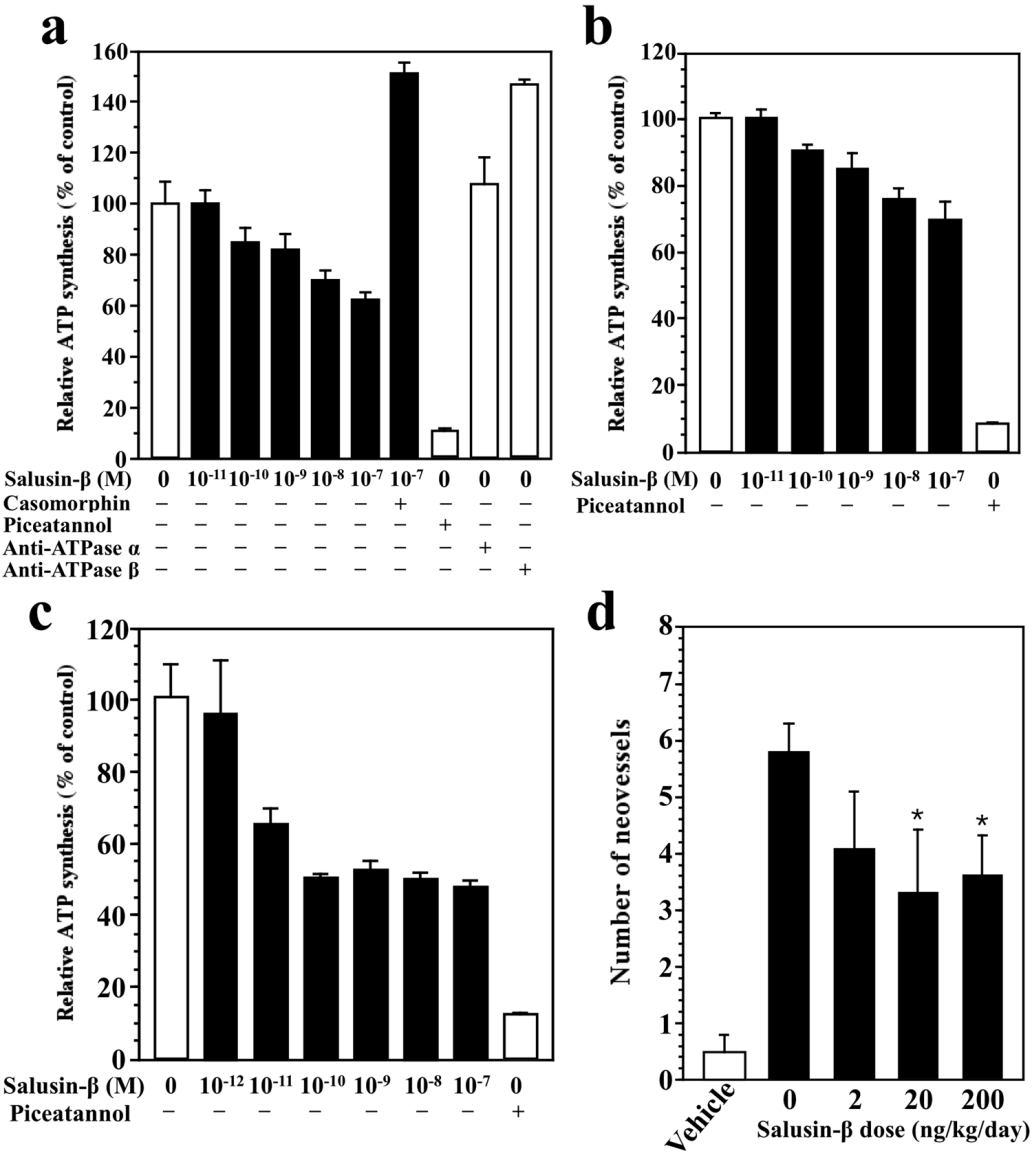
Suppression of cell surface ATP synthase activity and tumor-induced angiogenesis by salusin-β. (**a**–**c**) Salusin-β modestly and concentration-dependently inhibits cell surface ATP generation in HeLa cells (**a**) and hMVECs (**b**) cultured at pH 7.4. Pretreatment with casomorphin (10^−6^ M) abrogates the effect of salusin-β (**a**). The effect is more profound on hMVECs cultured at pH 6.7 (**c**) Extracellular ATP generation was measured by a bioluminescence surface assay following 20 s of incubation with medium in the presence and absence of ADP. Piceatannol is a stilbene phytochemical that inhibits F_1_F_o_ ATP synthase. (**d**) Sarcoma 180 cells were implanted into mouse dorsal air sacs and the indicated doses of salusin-β were administered subcutaneously once per day for 5 days. Tumor-induced angiogenesis in the skin was evaluated by counting the numbers of neovessels microscopically. Each column with a bar represents the mean ± SEM for eight mice. *P < 0.05 *vs*. control animals without salusin-β.

Endothelial cell surface ATP synthase is known to produce ATP, and its activity is inhibited by angiostatin at low pH^28^. We measured the functional activity of cell surface ATP synthase by quantifying the nucleotides generated in culture media using a CellTiterGlo™ luminescence assay. Salusin-β modestly inhibited cell surface ATP synthesis in HeLa cells (Fig. 6a) and hMVECs (Fig. 6b) at physiological pH in a concentration-dependent manner. Pretreatment with β-casomorphin antagonized the effect of salusin-β, while piceatannol, a known ATP synthase inhibitor, markedly inhibited ATP synthesis. Anti-ATP synthase β-subunit IgG increased the ATP synthase activity, whereas anti-ATP synthase α-subunit IgG had no effect (Fig. 6a). The effect on hMVECs was more profound at pH 6.7 (Fig. 6c), a condition in which angiostatin exerts an inhibitory effect on ATP synthesis. Salusin-β had no influence on ATP synthesis by rAECs or rPECs, which do not express the ATP-synthase β-subunit on their cell surface.

We then assessed the effect of salusin-β on the angiogenic response triggered by malignant tumor cells using the mouse dorsal air sac assay. Sarcoma 180 cells induce a robust angiogenic response in the otherwise poorly vascularized subcutaneous area when inserted into Millipore diffusion chambers and implanted into dorsal air sacs created in Crlj:CD1 mice^29,30^. Salusin-β was administered intravenously once daily for 5 days starting after the implantation of the Millipore chambers. Control animals were implanted with chambers with or without Sarcoma 180 cells and received ddH_2_O. At the end of the treatment, the animals were sacrificed. After removal of the chambers, the vascularization of the skin in contact with each chamber was observed microscopically. All tortuous microvessels longer than 3 mm, a characteristic feature of angiogenesis, were counted as neovessels. Salusin-β dose-dependently (2 and 20 ng/kg/day) inhibited the number of newly formed blood microvessels without inducing any toxic effects (Fig. 6d), thereby revealing an *in vivo* antiangiogenetic property. These observations suggest a role for salusin-β as a low molecular weight antiangiogenic peptide that mimicks the effect of angiostatin.

## Discussion

We have described a novel strategy to identify cell surface receptors for bioactive peptides. Conventional methods for identifying receptors using detergents to solubilize membrane proteins may modify the three-dimensional structures of the receptor proteins and affect ligand-receptor binding based on hydrophobic interactions. In the present study, use of conventional method identified a β-actin-profilin complex as membrane-associated salusin-β-binding proteins, but did not detect any cell surface receptors. In contrast, our new approach utilizing liposomes embedded with membrane proteins directly transferred from animal tissues successfully identified the cell surface targets of salusin-β. Cell surface receptors are enriched in emulsified proteoliposomes of <200 nm and can be retrieved using peptide ligands immobilized on a ligand support. Larger particle-sized liposomes not only prevent receptor-binding experiments and subsequent affinity purification, but also enclose the great majority of cell membrane proteins within the multi-layered liposomes. In contrast, rigid collection of particles smaller than 200 nm markedly increases the frequency of cell surface receptors as well as membrane-associated adaptors and lining proteins either penetrating or attached to the outer surface of lipid bilayers, while retaining their original binding capabilities toward ligands. Together with the results of our proof-of-concept studies, which demonstrated the ligand-binding capabilities of receptor proteins transferred from both animal organs and cultured cells, our data indicate a broad applicability of our proteoliposome methodology for identifying receptors for a variety of ligands irrespective of their receptor structures.

The present identification of the β-chain of ATP synthase as a principal cell surface target for salusin-β led to the discovery that salusin-β acts as an angiogenesis inhibitor. The ATP synthase β-subunit belongs to the F_1_ domain and had long been believed to be strictly confined to the mitochondrial inner membrane, but was later identified to serve as a cell surface receptor for apparently unrelated ligands, such as enterostatin^31^, β-casomorphin^32^, apolipoprotein A-I^33^, that modulate food intake, hypertension and lipoprotein metabolism, depending on the cell type and environment, by generating either ATP or ADP^34^. Angiostatin binds to the α-chain of ATP synthase of endothelial cell surface and inhibits endothelial cell migration and proliferation during tumor angiogenesis^35^. The present study has revealed that the endothelial ATP synthase β-subunit mediates the antiangiogenic properties of salusin-β, which has a far lower molecular weight than the potent angiogenesis inhibitor angiostatin.

Ligands that do not elicit common intracellular signaling pathways in cells overexpressing orphan GPCRs cannot be identified by reverse pharmacology strategies. Moreover, many bioactive peptides have been shown to utilize non-GPCR cell surface proteins as their functional receptors. Our present method for isolating novel receptors retrieves cell surface target proteins by utilizing their binding capabilities, thereby allowing the identification of the cell surface targets irrespective of the intracellular signaling pathways or types of receptors. Furthermore, in classical approaches that require lengthy processes to identify bioactive peptides, peptides showing tight and immediate binding to tubes, capillaries and tips, such as salusin-β, can easily be lost during the purification processes^25^. Therefore, an alternative approach to discovering ligands and receptors that are hard to identify by conventional methods has been desired. Our *in silico* ligand prediction method^8^, with which we identified the salusins, allows us to test the biological activities of a number of synthetic peptides predicted from genome and cDNA library resources, and enables us to focus on important peptides that elicit the most desired bioactivities. Further, we have successfully developed a new plasma native peptidomic technology that allows to efficiently remove plasma high abundant proteins to comprehensively identify circulating native peptides using mass spectrometry^9,10,36,37^. These approaches have already selected a number of plasma native peptides as putative bioactive peptide candidates and could be used to identify their cell surface targets with the current proteoliposome methodology.

## Methods

### Cell culture

Human monoblastic leukemia THP1 and U937, embryonic kidney HEK293 and neuroblastoma NB1 cells were purchased from the Health Science Research Resources Bank (Osaka, Japan). Human uterine cervical carcinoma cells, HeLa, and human umbilical vascular endothelial cells, hUVECs, were purchased from the Japanese Collection of Research Bioresources Cell Bank (Osaka, Japan). hMVECs were purchased from Kurabo (Osaka, Japan). Cells were cultured using appropriate medium and supplements recommended by the suppliers. rPECs and rAECs were prepared from the pulmonary artery and aorta of male Wistar rats, respectively, as described^8,38^.

### Preparation of liposomes embedded with membrane proteins

To prepare cell membrane protein fractions, 2 g of each animal organ suspended in 10 ml of 10 mM sodium phosphate (pH 7.4), containing DNaseI (70 U/μl) and 1 mM DTT was homogenized and centrifuged at 2500 rpm at 4 °C for 5 min. The supernatant was added to 12 ml of 40% saturated sucrose solution and centrifuged at 95,000 × *g* at 4 °C for 1 h. The membrane protein fractions layered between the two phases were collected and stored at −80 °C until use. Plasma membrane fractions were also obtained by homogenizing ~2 × 10^9^ cultured cells followed by density gradient centrifugation. Purified yolk lecithin (1.0 g; Asahi Kasei, Japan) and cholesterol (0.2 g; Wako Pure Chemical, Japan) were dissolved in 10 ml of chloroform, vacuum-dried at room temperature and reconstituted in 10 ml of 10 mM Tris-HCl (pH 7.4). The mixture was vigorously stirred to obtain a liposome suspension and stored at 4 °C until use. Five milliliters of 10 mM Tris-HCl (pH 7.4) was added to 50 mg of the cell membrane fractions, ultrasonicated using an Insonator 201 M™ (Kubota, Japan) on ice at 100 W for 15 min, and mixed with the liposome suspension. After three freeze-thaw cycles, the mixture was ultrasonicated again. To remove the large particles and collect particle-sized liposomes, the layer obtained from the mixture of the liposome suspension and cell membrane fractions was passed through a 200-nm polycarbonate membrane (Nucleopore; Whatman).

### Receptor binding studies using a variety of peptidic ligands

VIP and CRF were purchased from Sigma Genosys (Japan). The remaining synthetic peptides were obtained from Peptides Institute (Osaka, Japan). Reconstituted peptides (10 pmol each), either alone or in combination, were incubated with 0.5 mg of proteoliposomes for 1 h at 25 °C. The reaction mixture was extensively washed to eliminate unbound peptides by repeated ultracentrifugation at 10,000 × *g* and resuspension of the pellet with 10 mM Tris-HCl (pH 7.4). Peptides specifically bound to the proteoliposomes were eluted with 0.3 N HCl containing 0.1% Triton X-100. The molecular masses of the peptides in the eluates were determined using a ProteinChip Reader PBSIIc (Ciphergen Biosystems) or a BLOTCHIP (Protosera, Kobe, Japan), a target plate for MALDI-MS^39^, and subsequent analysis with an Ultra-flex II MALDI-TOF/TOF (Bruker Daltonics).

### Detection of uPA, interferon-γ and C5a receptors embedded in liposomes

Purified uPA was a gift from Mitsubishi Pharma Corp (Osaka, Japan). For binding studies using fluoroproteoliposomes, fluorescein-4-isothiocyanate (Dojindo, Tokyo, Japan) was labeled with phosphatidyl serine using the protocol recommended by the manufacturer and was then embedded with membrane proteins prepared from U937 cells pretreated with 150 nM PMA for 4 days. The fluoroproteoliposomes were then reacted with biotinylated uPA, interferon-γ or C5a immobilized on an avidinylated Sepharose 4B gel as a ligand support, and observed under visible and fluorescent light after extensive washing with PBS. For binding studies using FITC-labeled ligands, membrane proteins-embedded liposomes with larger particle sizes (~1000 nm) were allowed to bind to FITC-labeled uPA and human serum albumin (Sigma Genosys) and subjected to flow cytometry analysis (FACSCalibur; Becton Dickinson). Detection of uPA receptor retrieved from proteoliposomes using immobilized uPA employed protein G/anti-uPA IgG as a spacer on the plate for mass spectrometry. Briefly, protein G was crosslinked to the plate for mass spectrometry via a covalent bond onto which anti-uPA IgG was bound noncovalently. Membrane proteins-embedded liposomes prepared from PMA-stimulated U937 cells or simple liposomes without membrane proteins were overlaid onto the plate, incubated at 4 °C overnight and subjected to mass spectrometric analysis for the detection of uPA and/or uPA receptor. Control experiments utilized bovine IgG in place of anti-uPA IgG. To quantify the retrieval of uPA receptor bound to its immobilized ligand, cell membrane fractions (50 mg) extracted from PMA-stimulated U937 cells were mixed with 0.5 ml of alkaline phosphatase (400 U/ml) and liposomes. Proteoliposomes were allowed to bind to various amounts of uPA immobilized on 50 μl of Sepharose 4B gel. After elution, alkaline phosphatase was dissociated from the eluates by the addition of 100 μl of a buffer comprising 50 mM Tris-HCl, 2 mM MgCl_2_ and 1% Triton X-100. Alkaline phosphatase substrate was added, and the absorbances (405 nm/690 nm) were measured. The presence of uPA receptor was finally confirmed by mass spectrometry.

### Receptor isolation utilizing membrane proteins-embedded liposomes

Biotinylated ligands were immobilized on NHS Sepharose 4B and EAH Sepharose 4B, which both contain 6-aminohexanoic acid as a spacer molecule, and the two coupled gels were equilibrated with 10 mM Tris-HCl (pH 7.4). Ten milliliters of membrane protein fraction (5 mg protein/ml) was incubated with 50 μl of each ligand-coupled gel at 25 °C for 1 h and then ultracentrifuged at 10,000 rpm using an Optima™ TLX Personal Benchtop Ultracentrifuge (Beckman Coulter, CA). Reconstitution of the pellet with 10 mM Tris-HCl (pH 7.4) and ultracentrifugation were repeated three times to complete extensive washing. Membrane proteins bound to the immobilized ligands were eluted with 0.3 N HCl containing 0.1% TritonX-100. The elution process was repeated 3 times to retrieve as much ligand-bound protein as possible, and the collected eluates were resolved by SDS-PAGE. Silver-stained bands in the gel were dissected, incubated with trypsin at 35 °C for 20 h and subjected to LC-MS/MS analysis using a MAGIC 2002 (Michrom BioResources, CA) followed by analysis with a Q-TOF2 system (Waters Micromass, MA). The fragment masses were identified using MASCOT searches.

### Receptor purification using the conventional method

Membrane protein fractions (50 mg) prepared from rat heart and lung as described above was dissolved in 1 ml of 50 mM Tris-HCl (pH 7.4) containing 150 mM NaCl and 1% Triton X-100. After homogenization and centrifugation at 20,000 rpm at 4 °C for 40 min, 1-ml aliquots of the supernatant were overlaid onto NHS Sepharose 4B- and EAH Sepharose 4B-coupled gels already equilibrated with 50 mM Tris-HCl (pH 7.4) containing 150 mM NaCl and 0.1% Triton X-100. The gels were washed with 50 mM Tris-HCl (pH 7.4) containing 150 mM NaCl and 0.1% Triton X-100 and eluted with 0.3 N HCl containing 0.1% Triton X-100. The eluates were resolved by SDS-PAGE, and the silver-stained bands were analyzed as described above.

### Subcellular fractionation

Cultured cells were fractionated into plasma membranes (PM), high-density microsomes (HDM), low-density microsomes (LDM), and mitochondria/nuclei (M/N) using a previously described method^40^. Homogenization of the cells and subsequent handling of all fractions were performed at 4 °C in a buffer comprising 1 mM EDTA, 250 mM sucrose, 20 mM HEPES (pH 7.4), 1 mM PMSF and 1 mM aprotinin. The homogenate was centrifuged at 10,000 × *g* for 15 min, and the supernatant was centrifuged again at 20,000 × *g* for 60 min. The pellet was layered onto 1.12 M sucrose, 1 mM EDTA and 20 mM HEPES (pH 7.4) and centrifuged at 100,000 × *g* for 60 min, yielding a white interface band representing the PM fraction and a viscous brown pellet representing the M/N fraction. The supernatant obtained from the initial centrifugation was centrifuged again at 41,000 × *g* for 20 min, yielding a pellet designated as the LDM fraction.

### Immunoprecipitation

Quiescent or growing cells were stimulated with serum or agonists, respectively, at 37 °C for specified durations. Each stimulation was terminated by replacing the medium with ice-cold lysis buffer comprising 20 mM Tris-HCl (pH 7.4), 100 mM NaF, 10 mM sodium pyrophosphate, 5 mM EDTA, 5 mM sodium orthovanadate, 2 mM phenylmethylsulfonyl fluoride, 1% NP40 and 0.1 mg/ml aprotinin. After a brief centrifugation at 14,000 × *g* for 5 min, the supernatant was immunoprecipitated with protein G-agarose beads preadsorbed with the immunoprecipitation antibody (anti-profilin1/2 antibody, anti-salusin-β antibody or anti-PIP_2_ antibody) for 16 h at 4 °C. The immunoprecipitates were washed three times with assay buffer (50 mM HEPES pH 7.4, 150 mM NaCl, 5 mM EDTA, 1% NP40), solubilized in Laemmli sample buffer containing 2-mercaptoethanol, resolved by SDS-PAGE and transferred to nitrocellulose membranes (Amersham Pharmacia Biotech). After blocking with 5% milk, the membranes were treated with a primary antibody followed by a horseradish peroxidase-conjugated secondary antibody. Immunoreactive proteins were detected by enhanced chemiluminescence (Amersham Pharmacia Biotech). For repeated immunoblotting, the membranes were stripped with 62.5 mM Tris-HCl (pH 6.7) containing 2% SDS and 0.1 M 2-mercaptoethanol for 30–45 min at 50 °C.

### Confocal microscopy

HeLa cells plated on glass coverslips were deprived of serum for 24 h, pretreated with or without 10^−6^ M β-casomorphin 7 or enterostatin (Peptide Institute, Osaka, Japan) for 30 min, and incubated for 1 min after addition of indicated concentrations of salusin-β-FAM. Cells were then washed twice with PBS, fixed with 4% paraformaldehyde for 15 min and incubated for 60 min with specific antibody against the β-chain of ATP synthase (Molecular Probes, OR) diluted to 1:1000 in PBS and visualized with 30 min incubation with Alexa Fluor^*®*^ 594 secondary antibody (Molecular Probes). The nuclei were counterstained using DAPI Fluoromount-G^*®*^ (SouthernBiotech). Laser scanning confocal microscopy was performed using an LSM510 confocal microscope (Carl Zeiss, Jena, Germany) as described^41^.

### Cell surface binding of salusin-β

For quantification of fluorescent salusin-β-binding, HeLa cells cultured in non-coated 96 well black plates (Corning**®**) were plated to be confluent at the time of assay, replaced with serum starved media with or without the indicated concentrations of synthetic salusin-β in 0.05% NP40/HBSS for 30 min, and further incubated for 60 min after addition of FAM-labeled salusin-β in 0.05% NP40/HBSS. Cells were washed three times with PBS and fluorescence measured at an excitation wavelength of 485 nm and an emission wavelength of 535 nm using a SpectraMax M2 microplate reader (Molecular Devices). A specific cell-bound fluorescent peptide was calculated by subtracting non-specific adherent fluorescence arising from peptide material bound to the inside wall of the cylindrical polypropylene 96 well plate from the total fluorescence detected in each well.

### Measurements of cell surface ATP

Subconfluent hMVECs in 96-well plates were washed with serum-free medium, and treated with various concentrations of salusin-β, piceatannol (Sigma), anti-ATP synthase antibody or medium alone for 30 min at 37 °C under 5% CO_2_ to achieve pH_e_ 7.2 or 17% CO_2_ to achieve pH_e_ 6.7. The cells were then treated with ADP for 20 s, and the supernatants were immediately removed and assayed for ATP production by the CellTiterGlo™ luminescence assay (Promega)^42^.

### Mouse dorsal air sac assay

Malignant tumor cell-induced angiogenesis was assessed by Panapharm Laboratories (Kumamoto, Japan) as described^29,30,43^. Millipore chambers were prepared by covering both sides with Millipore filters (0.45-mm pore size) and filled with 5 × 10^6^ Sarcoma 180 cells suspended in 0.15 ml of PBS. The tumor cell-containing chambers were implanted into subcutaneous dorsal air sacs created in female Crlj:CD1 (ICR) mice aged 9 or 10 weeks (Charles River, Kanagawa, Japan) by injecting approximately 8 ml of air. The groups of mice treated with tumor cells were subcutaneously administered salusin-β at 0, 2, 20 or 200 ng/kg/day once per day for 5 days. Salusin-β (Peptide Institute, Osaka, Japan) was dissolved to 20 μg/ml in physiological saline containing 0.05% Tween 20 and further diluted to 0.2, 2 or 20 ng/ml for injection of 10 ml/kg/day. The mice in the non-tumor control group, which received chambers containing PBS in place of Sarcoma 180 cells, were administered vehicle alone. On day 5, the implanted chambers were removed from the subcutaneous fascia of the treated animals, and a black ring (Sanko Kagaku, Hiroshima) with the same inner diameter as the Millipore ring was placed at the same site. The angiogenic response was assessed under a dissecting microscope by counting the newly formed blood vessels of more than 3 mm in length within the area encircled by the black ring. The neovessels were morphologically distinguishable from the preexisting background vessels by their tortuous nature. The data were analyzed by a non-paired Wilcoxon rank sum test or a nonparametric Dunnett’s test where appropriate.

### Ethics statement concerning animal work

The authors confirm that all experiments were performed in accordance with relevant guidelines and regulations. Mouse dorsal air sac assays were carried out in accordance with procedures that were approved by the Animal Experimentation and Ethics Committee of Panapharm. All other animal experimental procedures were approved by the Animal Experimentation Ethics Committee of the Protosera Inc. and were performed in accordance with the guidelines and the regulations for its animal experiments.

## Electronic supplementary material

Supplementary Information
