# Supplementary material for: Identification of the salusin-β receptor using proteoliposomes embedded with endogenous membrane proteins
Source: Sci Rep. 2018 Dec 14;8:17865. doi: 10.1038/s41598-018-35740-6 (PMC6294790; doi:10.1038/s41598-018-35740-6)
Supplement: Supplementary file 1 — Supplementary Information [file 41598_2018_35740_MOESM1_ESM.pdf]

Supplementary Information

Identification of the salusin-β receptor using proteoliposomes embedded with endogenous membrane proteins

Masayoshi Shichiri[1], Daisuke Nonaka[2], Lyang-Ja Lee[2], & Kenji Tanaka[2]

[1]Department of Endocrinology, Diabetes and Metabolism, Kitasato University School of Medicine, 1-15-1 Kitasato, Minami-ku, Sagamihara, Kanagawa 252-0374, and [2]Protosera Inc., 4-3-22 Nishinakajima, Yodogawa-ku, Osaka 532-0011, Japan.

Corresponding author:

Masayoshi Shichiri

Department of Endocrinology, Diabetes and Metabolism

Kitasato University School of Medicine

1-15-1 Kitasato, Minami-ku, Sagamihara,

Kanagawa 252-0374

E-mail: shichiri@kitasato-u.ac.jp

Amino acid sequences of salusin-β and its related peptides.

**salusin-β**	**AIFIFIRWLLKLGHHGRAPP**
**salusin-β(4-20)**	**IFIRWLLKLGHHGRAPP**
**salusin-β(5-20)**	**FIRWLLKLGHHGRAPP**
**salusin-β(6-20)**	**IRWLLKLGHHGRAPP**

Hydrophobic amino acids are indicated in green characters.

Uncropped photographs of the protein blots shown in Figure 4.

Figure 4a

3-min exposure 30-min exposure

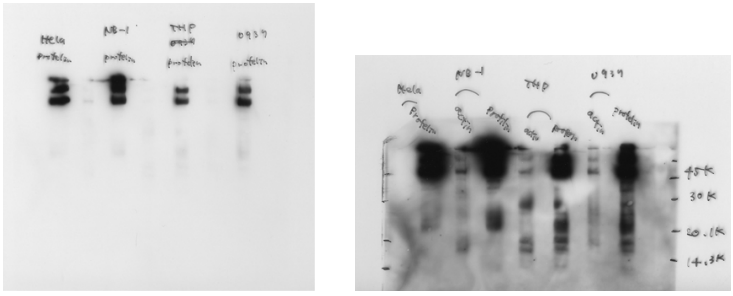

IP: anti-profilin 1/2 or anti-β-actin, Blot: anti-salusin-β

Figure 4b

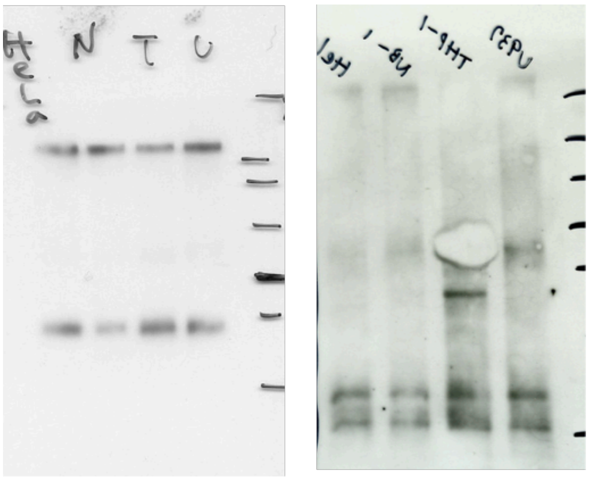

IP: anti-profilin 1/2 IP: anti-PIP_2

Blot: anti-salusin-β Blot: anti-profilin 1/2

Figure 4d

HeLa cells stimulated by 10% fetal bovine serum

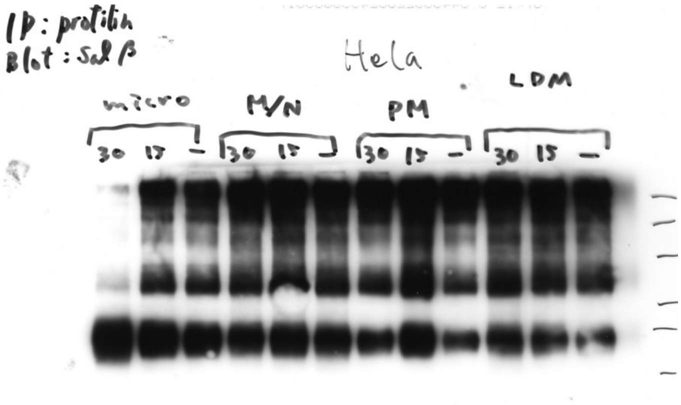

THP-1 cells stimulated by endothelin-1

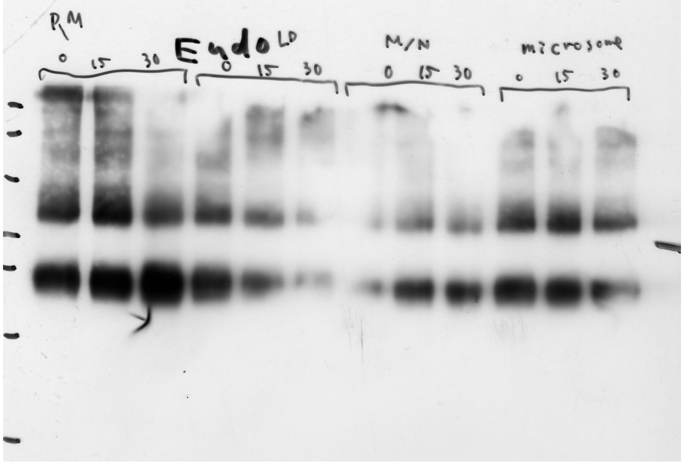

THP-1 cells stimulated by TNF-α

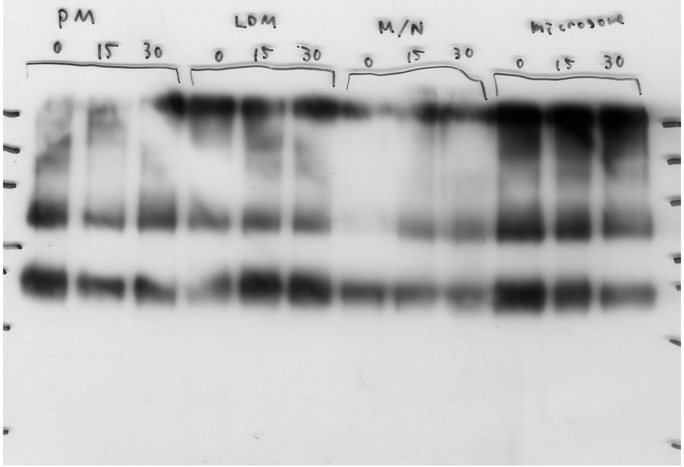

IP: anti-profilin 1/2

Blot: anti-salusin-β
